# Revealing the Complexity of Fatigue: A Review of the Persistent Challenges and Promises of Artificial Intelligence

**DOI:** 10.3390/brainsci14020186

**Published:** 2024-02-19

**Authors:** Thorsten Rudroff

**Affiliations:** 1 Department of Health and Human Physiology, University of Iowa, Iowa City, IA 52242, USA; thorsten-rudroff@uiowa.edu; Tel.: +1-(319)-467-0363; Fax: +1-(319)-355-6669; 2 Department of Neurology, University of Iowa Hospitals and Clinics, Iowa City, IA 52242, USA

**Keywords:** fatigue, artificial intelligence, machine learning, deep learning, ChatGPT, Claude

## Abstract

Part I reviews persistent challenges obstructing progress in understanding complex fatigue’s biology. Difficulties quantifying subjective symptoms, mapping multi-factorial mechanisms, accounting for individual variation, enabling invasive sensing, overcoming research/funding insularity, and more are discussed. Part II explores how emerging artificial intelligence and machine and deep learning techniques can help address limitations through pattern recognition of complex physiological signatures as more objective biomarkers, predictive modeling to capture individual differences, consolidation of disjointed findings via data mining, and simulation to explore interventions. Conversational agents like Claude and ChatGPT also have potential to accelerate human fatigue research, but they currently lack capacities for robust autonomous contributions. Envisioned is an innovation timeline where synergistic application of enhanced neuroimaging, biosensors, closed-loop systems, and other advances combined with AI analytics could catalyze transformative progress in elucidating fatigue neural circuitry and treating associated conditions over the coming decades.

## 1. Challenges and Opportunities for Progress in Understanding Fatigue

***Part I:* Lost in Complexity: A Review of the Key Challenges and Barriers Obstructing Progress in Understanding Fatigue**

Fatigue is an exceedingly common complaint that negatively impacts people’s health, productivity, and quality of life [1,2,3]. An improved understanding of biological drivers across chronic fatigue, cancer, and neurological, aging, and psychiatric conditions could enable treatments targeting dysfunction [4,5,6,7,8]. Decoding fatigue enigmas through emerging science is crucial for addressing this major source of disability burdening many patient groups [4,5,6,7,8]. However, fatigue remains a poorly characterized phenomenon from a biological standpoint despite its major personal and societal costs [1,2,3]. Progress in elucidating fatigue mechanisms has been hindered by several key challenges.

### 1.1. Lost in Translation: How the Subjective Language of Fatigue Complicates Mechanistic Understanding

Fatigue is a complex, subjective experience that manifests in myriad ways across mental, physical, and emotional domains [9,10,11,12,13,14]. The sensations of fatigue rely heavily on self-report and the description of subjective feelings of tiredness, exhaustion, lack of motivation, or difficulties with concentration and completing tasks [10,12,13]. This makes quantifying fatigue in precise, objective ways challenging [10,12,13]. Moreover, the terminology used to describe fatigue symptoms remains inconsistent, with terms like tiredness, exhaustion, weakness, and lack of energy or motivation often used interchangeably without clear consensus on the boundaries or distinctions between each sensation [13]. This linguistic variability in reporting subjective perceptions of fatigue creates barriers to clearly defining specific dimensions for measurement [10,12,13]. It also obscures understanding of how language used to express fatigue maps onto underlying biological mechanisms [11,12,13]. The inherent subjectivity of sensations grouped under “fatigue”, combined with fuzzy semantic delineations between associated terms, create difficulty in formulating consistent fatigue constructs for biological study. Research efforts become siloed by the specific term or phrasing used to describe sensations in a particular study rather than consolidating underlying mechanisms that may generalize across how fatigue is verbally expressed. This further hampers the aggregation of insights across the field. Addressing limitations in dealing with subjective terminology will be key to deriving coherent explanatory models of fatigue grounded in objective, generalizable metrics detectable across differences in self-reported language.

### 1.2. Addressing Key Translational and Interconnectivity Challenges

The experience of fatigue likely stems from dysregulation across a number of interconnected biological systems spanning different physiological levels, from cellular processes like mitochondrial energy generation all the way up to activation patterns in cognitive and sensory processing regions in the central nervous system [15,16,17,18]. The triggering of fatigue could arise from a combination of bottom–up energetic deficiencies creating bioenergetic stress on a cellular level, metabolic signaling irregularities, immune activation, and inflammation, as well as top–down neural signaling cascades linked to the perception of effort and motivation.

Disentangling the influence of these different systems with bidirectional associations adds major complexity to identifying primary causal pathways that drive the emergence of fatigue [15]. Interactions between cell energetics, cardiorespiratory factors, immune function, endocrine signaling, and sense of exertion mediated by central neural circuits create a dynamic network with many moving parts that can trigger fatigue. However, determining which nodes in this network represent key propagator mechanisms versus reactive components obscured by downstream effects presents challenges.

Isolating direct initiating factors in a complex, interconnected web has remained difficult, especially given that some propagators may lie at junctures between systems at different physiological scales [19]. Subtle energetic shortfalls at a cellular level triggering neural signaling may be obscured by the time they manifest as perceived fatigue [6,20]. This complexity means generic models of fatigue based on a single moderator have limited explanatory power. Instead, more sophisticated mapping of multifactorial influences, compensatory mechanisms, and system transitions will be essential to identify the primary drivers behind this complex phenomenon [21].

### 1.3. Bridging Individuality, Invasiveness, and Insularity Challenges

*Individuality.* There exists a large variability in how fatigue manifests between different people, even among those with similar health conditions or exposures to fatigue-inducing stressors. The triggers that induce fatigue, the time course of fatigue severity changes, the longevity of fatigue sensations, and the degree of functional disability can differ drastically across individuals [10,11,12,13]. For instance, some may experience more cognitive manifestations, like mental fog, while others feel only physical fatigue. Fatigue from sleep loss manifests more rapidly in some people [22]. Training loads provoking overtraining fatigue vary by fitness level [9]. Chronic illnesses often co-occur with fatigue, but with little consistency for predicting severity based on primary disease markers [10,11,12,13]. This substantial person-to-person variability in fatigue susceptibility and symptom patterns means generic models categorizing factors like duration of exposure to a stressor or activity level have limited power to explain, predict, or modulate fatigue. Explanatory frameworks may work reasonably well at population averages, but performance significantly degrades at an individual level. Blanket thresholds for triggers more often over- or under-estimate actual fatigue. There exists no “one size fits all” prescription for managing fatigue, unlike more stable phenomena. Accounting for individual differences likely requires assessing combinations of genetic predispositions, detailed sleep/circadian patterns, prior conditioning history, and other intrinsic physiological and neural traits interacting with situational demands. High-dimensional personalized profiles considering this web of modifiers operating uniquely in each person will likely be needed to enhance explanatory power for precise fatigue forecasting and effective interventions tailored to an individual.

*Invasiveness.* Obtaining objective measurements of fatigue is crucial for studying its biological basis [13]. Yet fatigue intrinsically complicates the implementation of gold-standard assays of central nervous system activity. The most definitive approaches for mapping neurophysiologic processes pertinent to feelings of tiredness, apathy, and reduced motivation rely on invasive methods inaccessible in awake, fatigued humans [23]. Techniques like extracellular single neuron recordings reveal detailed neural firing patterns but require direct brain parenchyma access in animals or neurosurgical implants, which are impossible in individuals experiencing natural fatigue [24,25,26].

Less invasive tools, like functional magnetic resonance imaging (fMRI) and positron emission tomography (PET), can capture whole human brain activity changes with fatigue but at low temporal resolution. Quantitative electroencephalogram (EEG) provides richer time-course data but limited spatial information. Small molecular biomarkers circulating at low levels that may catalyze fatigue symptoms are often below detection thresholds for standard assays [27]. Psychophysical measures of reduced muscular abilities, cognitive lapses, and decreased motivation during fatigue are easy to implement but rely on subjective self-reporting without confirming underlying biological correlates. This invasiveness limitation promotes heavier reliance on animal models to elucidate neurobiology [28,29,30]. Yet no single animal paradigm manages to recapitulate the full complexity of fatigue. Plus, human heterogeneity and drivers like motivation loss have no clear analogue. Thus, elucidating fatigue mechanisms through measurement remains seriously hindered by current technological and practical constraints around studying relevant signals in states where fatigue manifests.

*Insularity*. A significant barrier impeding progress in understanding fatigue is that associated research efforts have often occurred in silos segregated by disease area rather than consolidated across specialties to identify overarching biological principles. Because fatigue frequently co-presents across many illness contexts, like cancer, autoimmune disorders, infectious diseases, neurological conditions, and mood disorders, insights tend to accumulate in isolated pockets within these research niches [11,14]. Findings from one domain are often disconnected from complementary discoveries elsewhere. However, mechanistically parsing a complex phenomenon like fatigue occurring in association with diverse pathologies would greatly benefit from crosstalk across these specialties to recognize potential commonalities. Whether core propagators rely more on inflammatory signaling, metabolic irregularities, subtler mitochondrial dysfunction undetectable with standard assays, or disruptions in neurotransmission can remain obscured without discourse across groups studying these facets separately. Collaborative frameworks could powerfully catalyze discovery by connecting clues about dysregulation from cellular signaling analyses in one disorder to abnormal motor cortex activation patterns in another and blood markers flagged in a third disease [31,32,33]. Only through active consolidation of insights derived from compartmentalized investigations can researchers integrate fragments into unified explanatory models with translational potential. But, historical divisions have suppressed such vital cross-pollination.

### 1.4. Funding Prioritization Challenges

The lack of progress in understanding fatigue represents more than just conceptual scientific challenges. It is further perpetuated by consistent funding neglect from major governmental and nongovernmental sources allocating scientific research dollars [34,35]. Major initiatives have prioritized diseases to which fatigue is often secondary or ancillary despite fatigue’s independent debilitating effects. Its common co-occurrence relegates it to the status of an afterthought.

However, the immense detrimental quality-of-life impacts imposed by persistent sensations of draining exhaustion, mental fog, and depleted motivation regardless of root pathology warrant dedicated funding commensurate with its prevalence across society. With over a third reporting high fatigue levels at any given time across occupations and lifestyles, research focused explicitly on illuminating basic science around fatigue is disproportionately underfunded and attracts less interest in high-impact journals, limiting career appeal [34,35].

Without earmarked, large-scale funding initiatives targeting fundamental energetics, metabolomics, inflammatory profiling, and high-resolution neural mapping and modeling studies focused directly on fatigue itself rather than just a side effect of other syndromes, critical insights around pathogenesis and predictive markers are delayed. Prioritizing commensurate funding would accelerate knowledge development to match societal fatigue burdens.

### 1.5. Future Trends in Fatigue Research

As the understanding of fatigue as a complex, multi-system issue grows, research approaches are evolving to match this complexity. Key trends include utilizing big data and machine learning to find patterns across biological and behavioral data points [36], increased focus on the gut–brain axis and related mechanisms, like the impact of microbiome composition on fatigue [37], precision medicine initiatives to subtype different fatigue endophenotypes based on genetic, immune, and other biomarkers [38], and further research into lifestyle interventions like exercise, nutrition, and stress reduction that can potentially rebalance dysregulated systems underlying fatigue [39]. Technological advances in multi-domain biological data gathering, computer modeling, and data integration will likely accelerate these trends towards a big-picture, systems-based understanding of fatigue. This more holistic paradigm brings hopes of better diagnostics, subtyping of fatigue disorders, and potentially individualized or multi-modal therapies that can address root causes.

## 2. Domains of Application for Artificial Intelligence (AI) to Study Fatigue

**Part II: Forging New Frontiers: Opportunities for Artificial Intelligence and Neuro-technologies to Illuminate Fatigue Mechanisms**

Subjective fatigue spanning normal to pathological states presents a complex phenotype implicated across nearly all medical, psychological, and behavioral domains [4,5,6]. Any realm with multidimensional datasets capturing diverse aspects of fatigue experiences, biomarkers, and functional impacts holds promise as an application domain for AI techniques.

Chronic conditions like multiple sclerosis, cancer, Parkinson’s disease, and autoimmune disorders that frequently involve problematic fatigue provide relevant physiological data resources to employ machine learning algorithms. By modeling patterns across reported symptoms, blood panels, genetics, neuroimaging, and task performance metrics, predictive subtypes not constrained by clinical criteria could emerge [40]. Electronic health records data also offer a training field for neural networks to classify who will develop disabling exhaustion.

Wellness technology and wearable biosensors that track individual variability in fatigue experiences over time may enable personalized phenotyping through deep learning timeseries analysis [37]. Multimodal data fusion could illuminate circadian, metabolic, and lifestyle contributors. Reinforcement learning agents that suggest activity pacing and other interventions through virtual trials may also improve quality of life.

Broad research infrastructure development and participatory partnerships are imperative to compiling shareable corpora spanning the extensive heterogeneity within each domain’s fatigue manifestations [41]. Scientific working groups helping coordinate scope and quality standards for training sets will unlock fuller benefits across the spectrum until subjective exhaustion becomes objectively characterized through AI tools [42].

### 2.1. Illuminating the Complex Phenotype of Fatigue with Integrative AI

The subjective experience of fatigue involves an intricate web of physiological, molecular, neural, psychological, and environmental factors that vary significantly across individuals and circumstances [6,24]. This complexity and this heterogeneity have profoundly hindered progress in elucidating mechanisms, biomarkers, and interventions related to fatigue [43]. Research progress has also been limited by overspecialization and the challenges involved in systematically consolidating multimodal biomedical data to derive integrated explanations. Emerging techniques in AI, including machine learning and deep learning applied to aggregating complex datasets, offer promising analytic advances to overcome these obstacles [30,44]. Machine learning and deep learning are very much considered core parts of the field of artificial intelligence. Advances in these areas are driving much of the current excitement and breakthroughs in AI [45,46,47,48].

Machine Learning Approaches. Supervised machine learning involves computational algorithms trained to recognize multivariate patterns that differentiate classes and predict outcomes within multidimensional datasets [49]. By applying predictive modeling to diverse physiological datasets integrating molecular profiles, neural signals, reported symptoms, and task metrics from individuals exhibiting both shared and distinct aspects of fatigue, machine learning provides the potential to elucidate patterns of similarities and differences linked to mechanisms [44]. Studying fatigue via endocrine, immunological, genetic, self-reported, and neuroimaging data concurrently within individuals is crucial considering the evidence that fatigue comorbidities have interconnected influences [43]. Diverse neural network architectures, boosting methods, support vector machines, and clustering algorithms have all shown utility for handling heterogeneous, high-dimensional data and high-order feature interactions characteristic of fatigue phenotypes [40,44].

Deep Learning Techniques. As an evolution of multilayer neural networks, deep learning uses multiple processing layers to transform input data into hierarchical, abstract representations [49]. Applied directly to raw neuroimaging data, such as functional MRI, EEG, and PET scans related to fatigue, deep learning offers opportunities to extract patterns that allow for the classification of fatigue levels or phenomenological subtypes [44]. Generative deep models also facilitate imputing missing data modalities, which is valuable for integrating disjointed findings across incomplete historical studies. Translation techniques allow model interpretation to generate data-driven, functional hypotheses regarding neural dynamics and connectivity linked to fatigue experiences, mechanisms, and interventions [40].

Research Infrastructure and Priorities. Realizing the potential synergies afforded by applying artificial intelligence techniques to illuminate mechanisms underlying fatigue relies upon constructing collaborative research infrastructures for integrated phenotypic assessment, open science data sharing, enhanced funding, and improved regulatory alignment [50]. Multidisciplinary consortiums focused on deep patient phenotyping for precision medicine have provided frameworks encompassing virtual datasets integrating genomics, multiomic analysis, personal health record data, wearable readings, and ecological phone assessments that could be adapted to produce data resources for training integrative fatigue algorithms at scale [51]. Scientific working groups and federal harmonization initiatives also provide approaches that could incentivize and structure the formation of much-needed collaborative repositories optimized for applying multifaceted AI capabilities to transform understandings of fatigue’s complex biology.

### 2.2. Utilizing AI to Decode Fatigue’s Multifaceted Complexity

Fatigue poses a massive challenge for biomedicine as it is implicated across nearly all disease states yet still enigmatic in its phenomenology and biological origins [10,11,12,13]. Both the subjective multidimensionality of fatigue experiences and the interconnectivity of influencing factors defy reductionist explanatory models. Advanced AI presents versatile analytic approaches well-suited to mapping complex systems [52]. Techniques like deep neural networks, reinforcement learning, and generative adversarial networks applied to heterogeneous fatigue data can derive new biomarkers, phenotypes, and treatment targets.

Multilayer feedforward neural networks and convolution networks can pinpoint patterns in diverse biomarker data that connect to fatigue. By training on combined physiological datasets encompassing gene expression, clinical lab tests, cytokine panels, EEG readings, and patient reports, high-dimensional fatigue “fingerprints” can emerge that classify severity and subtype [44]. Preprocessing via principal component analysis and upset plotting informs key feature contributions. Recurrent models analyze sequential biomarker data as well, better capturing dynamic aspects.

Unsupervised approaches like k-means clustering applied to consolidated fatigue datasets discover potential phenotypes beyond patient-reported case definitions [40]. Reinforcement learning agents iteratively simulate treatment responses, rapidly screening hypothetical targets. Generative adversarial networks synthesize artificial yet realistic fatigue-related data to power deep neural network training [53].

Multimodal fatigue recordings parsed via signal processing and sensor fusion chronicle fluctuating real-world experiences [54]. Probabilistic graphical models characterize conditional dependencies among variables, inferring likely upstream and downstream mechanisms. Hybrid models should integrate bottom–up signal analytics with top–down systemic modeling to fully decode fatigue complexity as attempts narrow knowledge gaps.

### 2.3. Machine Learning for Pattern Discovery in the Fatigue Phenotype

Subjective fatigue spanning healthy to pathophysiological states involves dynamic interactions between physiological systems, genetics, lifestyle factors, and psychological processes—a complex phenotype still poorly characterized [43]. Machine learning offers versatile analytic approaches to illuminate multivariate patterns and inherent structures in such heterogeneous high-dimensional data that evade human examination [55]. Both supervised and unsupervised techniques applied to diverse fatigue-related datasets can derive novel insights.

Multilayer neural networks and ensembles of decision trees capture nonlinear relationships and high-order interactions between variables predictive of fatigue level and trajectory based on biochemical, immunologic, imaging, genomic, and patient-reported data [44]. Support vector machines efficiently find optimal boundaries that separate distinct fatigue subtypes or stages. Recursive feature elimination and principal component analysis preprocess unwieldy data.

Unsupervised learning is crucial for discovering natural fatigue phenotypes from bottom–up data without imposing existing classification constraints [40]. Algorithms like non-negative matrix and tensor factorization help identify parts-based biomarker subsets and co-occurrence patterns. Gaussian mixture models estimate the number of distinct biological processes models encompass.

Top–down hybrid approaches then integrate data-driven machine learning components with expert knowledge in causal probabilistic graphs and process-based simulations to further disentangle mechanisms [56]. Machine learning offers empirical fuel for decoding fatigue’s complexity; optimizing its potential relies on multidisciplinary data integration.

### 2.4. Deep Learning for Data-Driven Fatigue Phenotyping

The subjective, multifaceted nature of fatigue poses barriers to quantification that have hindered research progress for this critical quality-of-life factor across disease states [10,11,12,13]. Deep learning methods offer advantages in analyzing complex patterns within multidimensional data to empirically derive fatigue phenotypes and neural representations.

Deep neural networks compose layered transformations that uncover hierarchical non-linear feature representations within raw data, like neuroimaging scans [50]. Convolutional architectures analyze spatiotemporal patterns in functional MRI, EEG, and MEG signals that classify fatigue level and subtype without relying on predefined regions of interest [40]. Recurrent networks capture temporal dynamics in sequential biomarker data that characterize fatigue fluctuations.

Unsupervised techniques are crucial for discovering intrinsic patterns. Autoencoders compress then reconstruct high-dimensional omics data to capture key fatigue-related genetic and molecular factors in compact representations [44]. Generative adversarial networks can mitigate limited dataset sizes by synthesizing diverse, realistic fatigue biomarker distributions [53]. Self-supervised pretraining predicts masked aspects, extracting useful features before fine-tuning on labeled data.

Integrating deep learning components with multimodal data aggregation and expert-derived constraints within explanative modeling frameworks capitalizes on respective strengths [52]. The versatility of deep learning offers tangible progress towards data-driven fatigue phenotyping.

### 2.5. Towards Valid AI Models of Fatigue: The Importance of Multifaceted Evaluations for Reliable Scientific Progress

A rigorous framework for evaluating proposed AI methods is crucial for reliable progress in leveraging techniques like machine learning (ML) for gaining insights into complex health and behavioral phenotypes like fatigue [43]. Quantitative cross-validation approaches that assess the model’s skill at making predictions out of sample on new data promote generalizability [40]. However, due to the challenges of sufficiently enrolling cohorts spanning the heterogeneity of fatigue experiences, evaluations of ML algorithms aimed at classifying or stratifying fatigue should report metrics of predictive performance across datasets that represent realistic inter-study variability [57].

Additionally, testing across subsets of input data modalities (e.g., symptoms, wearable sensor streams, neuroimaging, multiomic, etc.) via sensitivity analysis is important for characterizing robustness [58]. Qualitative inspection of models’ results by domain experts, explanation of the biological plausibility of predictive patterns, and error analysis can mitigate risks of ML models simply learning statistical rather than more valid relationships from complex fatigue data [59]. Simulations that help assess concordance between emerging databased AI models and current theory-based knowledge about crash, post-exertional, and chronic fatigue mechanisms also remain crucial for synergistic scientific progress [60]. Future evaluations should utilize nuanced, multifaceted approaches to establish valid, complex AI methods that enhance understanding of fatigue across conditions.

### 2.6. Barriers to Illuminating Fatigue Mechanisms with AI

While AI methods like ML and DL offer advantages in analyzing multidimensional data to empirically phenotype fatigue states, substantial barriers constrain current capabilities. Fundamentally, the subjective and temporally dynamic nature of fatigue impedes assembling optimally large, heterogeneous, high-quality training datasets encompassing the diversity of influential biological and experiential factors [10,11,12,13]. Small sample sizes prone to sampling bias propagate risks of AI models achieving apparently strong predictive performance by capitalizing upon spurious correlations that fail to generalize or accurately reflect complex multisystem fatigue mechanisms [61].

Even with attempts to integrate multimodal data, ML algorithms exploring patterns spanning self-reports, wearable biosensor streams, genetics, lab tests, and neuroimaging may simply inherit and perpetuate the limitations of the underlying datasets and theories they draw upon. Their black box complexity also constrains model transparency and biological interpretability compared to explainable theory-driven models [46]. While DL techniques offer promise in processing raw physiological signals, validating their fidelity becomes challenging without established characterizations of fatigue pathophysiology to reference [52].

Progress requires sharing analytical techniques with an expansive, coordinated research agenda encompassing foundational science, continued ontology development, and a high-quality phenotypic data infrastructure. Multidisciplinary teams should guide the appropriate implementation of AI tools, the contextual interpretation of the findings, and bidirectional exchanges with basic researchers to overcome current barriers impeding AI-augmented illumination of fatigue’s origins and impacts [62].

## 3. How to Develop an AI Chatbot for Research

Advances in conversational AI offer researchers opportunities to create optimized digital interfaces for nearly all aspects of the scientific process. A major potential application of these systems involves natural language virtual agents or “chatbots” designed to interact directly with human subjects and study participants via flexible dialogue, replacing or augmenting traditional hand-coded online survey tools [63]. For example, a chatbot could be leveraged to enhance the recruitment of representative subject samples, the fine-tuning of eligibility screening for specialized protocols, the administration of standardized questionnaires [64], or even intervention delivery and progress tracking. However, successfully implementing chatbots in research requires methodical development and evidence-based optimization.

The chatbot design process should be initiated by explicitly defining the intended purpose and target population [65]. If aiming to act as an intelligent screener for a clinical trial on depression treatments, as one illustration, the system must encompass domain-specific vocabulary and appropriate tone given mental health considerations, and it must include validated instruments for assessing current symptoms and prior diagnoses. Output from all system dialogues must store variables in formats optimized for analysis as well. With a clear understanding of how chatbot capabilities will map to research goals, conversational frameworks can be outlined that logically guide users from open-ended welcome greetings to specific prompts gathering required data elements. Extensive usability testing, ideally in the ultimate target cohort, is critical before progressing [63].

Natural language processing methods, like bidirectional encoder representations from transformers (BERT), enable training the AI to reliably interpret diverse free-text responses from users rather than relying solely on restrictive multiple choice options [51]. These techniques require curating extensive repositories of raw language examples that the system may encounter associated with labels describing the intended meaning to “learn” contextual intent recognition.

The tuned language comprehension model can then be embedded within a modular conversational architecture encompassing dynamic response generation components [65]. Analytics should track all system interactions, and the entire pipeline must operate on secure platforms to safeguard confidentiality. Launch constitutes only the beginning—chatbot intelligence further evolves continuously in research through participant feedback, empirical performance benchmarking, and iterative improvements towards optimally addressing study aims.

### 3.1. From a Literature Review to Lab Work: Claude and ChatGPT as Collaborators in Fatigue Research

Advancing understandings of fatigue mechanisms and treatments represents an important scientific challenge requiring innovative approaches. AI tools offer promising capabilities to help review existing findings, generate fresh theoretical insights, plan rigorous studies, and formulate ideas for high-impact research projects [66,67]. Specifically, conversational agents like Anthropic’s Claude [68] and OpenAI’s ChatGPT (version 3.5) [69] permit direct querying and dialoguing with sophisticated language models. These systems have been trained on immense textual datasets, allowing them to interpret questions, synthesize information into well-structured responses, and maintain coherent multi-turn conversations. By enabling fluid interaction with expansive models of knowledge and discourse, Claude and ChatGPT empower researchers to leverage these AI systems as collaborative partners in pushing fatigue science forward.

The following explores productive applications of these conversational tools for literature reviews, hypothesis development, study design, perspective drafting, and grant proposal ideation to unravel the multifaceted phenomenon of fatigue through emerging techniques. The key premise is that thoughtfully guiding AI conversations can accelerate human discovery efforts. Scientists who leverage Claude and ChatGPT as part of their process will amplify true knowledge advances about fatigue’s mechanisms and treatments [70].

### 3.2. Literature Knowledge Synthesis

Conducting exhaustive literature reviews is fundamental but time-consuming for scientists. Claude and ChatGPT can serve as research assistants in compiling relevant publications on fatigue by rapidly reviewing key details in multiple papers, highlighting open questions around biomarkers and mechanisms, identifying seminal studies, and synthesizing core discoveries across various models and experimental approaches. Their ability to connect findings across disciplinary silos could allow them to suggest novel perspectives. Researchers can supply queries, areas of focus, and clarification questions to efficiently extract current knowledge status on issues of interest from these AI tools [70,71].

### 3.3. Hypothesis Generation

True scientific advances require creative leaps to formulate testable explanatory hypotheses [72]. Claude and ChatGPT facilitate such conceptual ideation by allowing for the rapid iteration of theories based on probing their expansive knowledge of potential fatigue contributors. For example, asking them to speculate on ways nutritional factors, microbiome changes, or subclinical infections might trigger fatigue could yield promising new angles. Researchers can integrate the most intriguing concepts from these conversations into draft models fleshing out putative causal chains, influences, and interactions driving fatigue’s onset and progression [73]. Such AI-assisted theory building can crystallize high-potential hypotheses to prioritize for empirical examination.

### 3.4. Study Design

Carefully constructed studies are necessary to evaluate speculative hypotheses, but designing research with sufficient power for conclusive inferences poses challenges [71]. Dialoguing with Claude and ChatGPT on study considerations can provide constructive input [57,58,59,74]. Researchers can describe nascent study ideas and have conversations examining issues like useful comparison groups, the feasibility of recruiting certain clinical populations, biomarker and endpoint selection to increase sensitivity for hypothesis tests, and modeling approaches to extract maximal insights from the collected data. The systems’ broad knowledge informs estimates of how adjusting factors like sample size, methods, and measurement strategies may strengthen the experimental foundation for testing mechanistic hypotheses about fatigue’s etiology [74,75].

### 3.5. Perspective Commentary Ideation

In addition to driving novel fatigue research, AI tools can assist scientists in consolidating current knowledge into perspective summaries for the research community. For instance, Claude can rapidly generate initial drafts of sections reviewing how techniques like predictive analytics, closed-loop stimulation systems, or pattern recognition on multimodal biomarker data might clarify understanding or lead to interventions for fatigue syndromes. Researchers can provide the framework and direction for such sections during the drafting process. The commentaries can outline promises and challenges around emerging approaches while synthesizing existing evidence and unknowns regarding mechanisms targetable for advanced analytics. This output helps update the broader scientific field on the frontier of progress.

### 3.6. Grant Proposal Planning

Research projects aimed at cracking open new dimensions to unravel fatigue’s etiology will require funding support. Claude and ChatGPT allow for collecting promising seed ideas for grant proposals during conversational sessions focused on how applying cutting-edge data science capabilities could accelerate knowledge growth [66,67,69]. Researchers can query the systems on goal setting for projects to uncover fatigue propagators, high-priority research gaps limiting treatment advances, and how new analytical methods offer opportunities. Gathering these idea fragments can help organize critical pieces of an impactful grant application, including laying out important questions, grounding technique selections in the literature, communicating significance for projected gains, and painting a vision for innovation in fatigue elucidation using AI capabilities.

In summary, progress in elucidating fatigue represents a challenging but crucial area of biomedical research requiring marshaling emerging technologies like AI. Conversational agents provide powerful tools to aid human researchers throughout conceptualization, planning, and theorizing around high-impact science directions. Effectively leveraging Claude and ChatGPT as collaborative thought partners via guided dialogue can expand progress in disentangling the complex web of mechanisms driving fatigue [66]. Scientists who productively harness these AI systems as part of their discovery process will amplify true knowledge advances.

### 3.7. Limitations of AI Chatbots in Science

AI chatbots, such as Claude and ChatGPT, have seen rapid advances in recent years, demonstrating impressive conversational abilities. However, their application within scientific domains remains limited due to a number of key challenges and limitations.

A fundamental limitation of chatbots is their lack of true semantic understanding behind conversations [70]. While advancements have been made in language modeling to generate coherent responses, chatbots still mostly rely on surface-level pattern matching rather than comprehending linguistic meaning and context. This restricts their applicability for science fields reliant on conceptual knowledge and reasoning. Simple information retrieval cannot replace the deep mastery and critical analysis expected from scientific experts [70,71].

Relatedly, chatbots currently lack human-level reasoning abilities for logical inference, abstraction, and critical evaluation [71,72]. Without capacities for logical deduction, scientific scrutiny, and identification of false claims or questionable conclusions, chatbots cannot robustly assist scientists or produce novel insights [71,72]. Attempts to mimic scientific hypothesis testing and analysis often resort to fabricated responses lacking substantive meaning [70,71,72].

While chatbots can display surface-level domain knowledge for constrained topics, their scientific understanding remains exceptionally narrow and brittle compared to human experts [71,72]. Restricted knowledge is prone to fast deterioration outside strict parameters, unlike the robust conceptual mastery of scientists. Additionally, language model deficiencies cause inconsistent, contradictory, and factually incorrect outputs when chatbots lack familiarity with queried topics. This unreliable performance presently renders autonomous chatbot participation in advanced research infeasible [71,72].

In summary, currently, deficiencies in comprehension, reasoning, and critical thinking and unstable mastery of scientific principles severely curb chatbots’ capabilities for reliably contributing to impactful science. With ongoing progress in AI, some constraints may relax, but human guidance and judgment will remain essential for responsible application in sensitive scientific domains for the foreseeable future.

## 4. Illuminating Fatigue’s Neural Circuitry: An Emerging Technologies Time Travel

Fatigue remains a perplexing phenomenon with unclear neurobiological underpinnings and mechanisms operating across interconnected physiological systems. Advancing diagnostics and treatments for fatigue conditions requires decrypting its complex neural signaling cascades and decoding dynamic signatures manifesting in neuroimaging data. Emerging techniques like mobile brain imaging, high-density EEG, multimodal neural fusion models, closed-loop stimulation systems, and brain–computer interfaces integrated with AI promise capabilities that could revolutionize understandings of fatigue’s neural circuitry. By interweaving neurotechnologies and advanced analytics, scientists may unlock fatigue’s mysteries. Here, we will explore an envisioned time travel of innovations that could transport fatigue science across the frontiers of progress over the coming century.

### 4.1. The Near Future: Tracking and Predicting Neural Fatigue Signals

Within the next decade, wearable mobile EEG–fMRI fusion modeling will enable the tracking of real-time neural correlates and connectivity changes associated with fatigue emergence and recovery at an individual level. Meanwhile, high-density EEG neural networks leveraging multivariate pattern analysis will achieve accurate personalized fatigue level predictions and risk forecasting. Such AI-powered brain mapping will deliver biomarker feedback to guide timely interventions.

### 4.2. Mid-Century: Multimodal Neural Signatures for Subtyping and Modulating Fatigue

By 2050, integrated diagnostics combining genomic, proteomic, and metabolic neural imaging will facilitate data-driven subtyping of chronic fatigue conditions. This will permit matching subtype-specific treatments to disease endotypes based on their underlying biology. In parallel, closed-loop stimulation systems harnessing real-time fMRI integrating EEG inputs will have demonstrated efficacy for AI-optimized modulation of fatigue circuitry.

### 4.3. The 22nd Century: Pre-Empting and Permanently Remediating Fatigue

As the 2100s approach, nanoscale sensor networks with wireless data integration will enable multi-organ snapshots of bioenergetic processes predicting incipient fatigue hours before symptoms manifest. Brain–computer interface symbionts with neural feedback and reinforcement learning will continuously optimize cognitive/physical energy levels for enhanced performance. Genetic anti-fatigue therapies will permanently recalibrate the molecular pathways underlying fatigue based on an individual’s neural imaging profile.

### 4.4. Convergence Driving Breakthroughs

By strategically coordinating the rollout of enhanced neuroimaging, analytic modeling, and simulation innovations in the coming decades, researchers can catalyze synergistic advances, equipping them to finally decipher fatigue’s neural code. The future integration of high-fidelity fatigue brain mapping with analytics and interventions looks very exciting.

## 5. Summary

**Part I** reviews persistent challenges obstructing progress in understanding complex fatigue’s biology. Despite fatigue’s immense personal and societal burdens, fundamental mechanisms driving the development and persistence of sensations like exhaustion, mental fog, and motivation loss have remained enigmatic. By consolidating an updated perspective on obstacles spanning the subjective multidimensionality of fatigue experiences, molecular-to-neural interconnectivity, individual variability, limitations of physiological sensing techniques, research/funding insularity, and communication barriers, this review freshly reveals the breadth of impediments obstructing impactful scientific advances and underscores both a novel urgency and a timely opportunity. Difficulties quantifying subjective symptoms, mapping multifaceted mechanisms, accounting for individual variation, enabling invasive sensing, overcoming insularity, and funding neglect are discussed.

**Part II** explores how emerging techniques like mobile brain imaging, biosensor analytics, advanced modeling approaches, and translational partnerships offer new opportunities to synergistically accelerate progress—either incrementally via status quo pathways or rapidly through strategic coordination with collaborative digitized data infrastructure.

Exciting opportunities exist for employing artificial intelligence, especially machine and deep learning, to integrate personalized multi-omics profiles with behavioral and neural features. Creative modeling strategies are highlighted for optimally utilizing these advances to overcome long-standing individual variability, invasiveness, and communication challenges surrounding fatigue investigation. Potential applications, like pattern recognition of complex physiological signatures as more objective biomarkers, predictive modeling capturing personal nuances, consolidating disjointed findings via data mining, and simulation to explore interventions, are discussed. Envisioned is an innovation timeline where the application of enhanced technologies combined with AI analytics could catalyze transformative progress in elucidating fatigue’s neural circuitry and treating associated conditions over the coming decades. Researchers strategically leveraging modern tools and shared data approaches will drive this new era of understanding.
